# Patterns of conventional and complementary non-pharmacological health practice use by US military veterans: a cross-sectional latent class analysis

**DOI:** 10.1186/s12906-018-2313-7

**Published:** 2018-09-05

**Authors:** Melvin T. Donaldson, Melissa A. Polusny, Rich F. MacLehose, Elizabeth S. Goldsmith, Emily M. Hagel Campbell, Lynsey R. Miron, Paul D. Thuras, Erin E. Krebs

**Affiliations:** 1 Minneapolis VA Health Care System, One Veterans Drive, Minneapolis, MN 55417 USA; 2 University of Minnesota Medical Scientist Training Program, Minneapolis, MN 55455 USA; 3 Division of Epidemiology and Community Health, University of Minnesota School of Public Health, Minneapolis, MN 55454 USA; 4 University of Minnesota Medical School, Minneapolis, MN 55455 USA

**Keywords:** Complementary integrative health, Alternative medicine, Non-pharmacological therapies, Latent class analysis, Veterans

## Abstract

**Background:**

Non-pharmacological therapies and practices are commonly used for both health maintenance and management of chronic disease. Patterns and reasons for use of health practices may identify clinically meaningful subgroups of users. The objectives of this study were to identify classes of self-reported use of conventional and complementary non-pharmacological health practices using latent class analysis and estimate associations of participant characteristics with class membership.

**Methods:**

A mailed survey (October 2015 to September 2016) of Minnesota National Guard Veterans from a longitudinal cohort (*n* = 1850) assessed current pain, self-reported overall health, mental health, substance use, personality traits, and health practice use. We developed the Health Practices Inventory, a self-report instrument assessing use of 19 common conventional and complementary non-pharmacological health-related practices. Latent class analysis was used to identify subgroups of health practice users, based on responses to the HPI. Participants were assigned to their maximum-likelihood class, which was used as the outcome in multinomial logistic regression to examine associations of participant characteristics with latent class membership.

**Results:**

Half of the sample used non-pharmacological health practices. Six classes of users were identified. “Low use” (50%) had low rates of health practice use. “Exercise” (23%) had high exercise use. “Psychotherapy” (6%) had high use of psychotherapy and support groups. “Manual therapies” (12%) had high use of chiropractic, physical therapy, and massage. “Mindfulness” (5%) had high use of mindfulness and relaxation practice. “Multimodal” (4%) had high use of most practices. Use of manual therapies (chiropractic, acupuncture, physical therapy, massage) was associated with chronic pain and female sex. Characteristics that predict use patterns varied by class. Use of self-directed practices (e.g., aerobic exercise, yoga) was associated with the personality trait of absorption (openness to experience). Use of psychotherapy was associated with higher rates of psychological distress.

**Conclusions:**

These observed patterns of use of non-pharmacological health practices show that functionally similar practices are being used together and suggest a meaningful classification of health practices based on self-directed/active and practitioner-delivered. Notably, there is considerable overlap in users of complementary and conventional practices.

**Electronic supplementary material:**

The online version of this article (10.1186/s12906-018-2313-7) contains supplementary material, which is available to authorized users.

## Background

Non-pharmacological therapies and self-management practices include approaches considered “conventional,” such as exercise and manual physical therapy, and those considered “complementary,” such as yoga and chiropractic manipulation. These health practices are commonly used by American adults [1, 2] and are recommended for prevention and management of a wide variety of illnesses, including, for example, hypertension [3], chronic musculoskeletal pain [4–6] and depression [7].

Prior studies have characterized users of individual practices [8] and types of practices [9], typically by examining practices categorized according to expert opinion [10] or researchers’ interests. Categorization of non-pharmacological health practices can vary widely between studies. Although most studies distinguish between complementary and conventional practices, the utility of this distinction is unclear. Evidence suggests people do not use individual complementary practices in isolation, but rather in combination with other complementary and conventional modalities [11, 12]. Certain health practices may cluster in meaningful ways that could define functionally meaningful categories of non-pharmacological health practices. Understanding factors associated with these patterns could help tailor care for patients and increase uptake of evidence-based practices.

The aims of this study were 1) to identify distinct patterns and categories of health practice use through latent class analysis; and 2) to estimate associations between latent class membership and sociodemographic, psychological, behavioral, and pain characteristics of users. We also describe development of a self-report tool for assessing health practice use.

## Methods

### Development of Health Practices Inventory

We developed the Health Practices Inventory (HPI) to facilitate valid self-report assessment of non-pharmacological therapies and health-related practices, including complementary and conventional approaches. Our primary goal was to evaluate use of therapies and practices for management of chronic pain; however, the inventory was designed to be broadly applicable beyond pain treatments. An initial list of 28 therapies and practices with brief definitions was developed after review of the National Health Interview Survey Complementary and Alternative Medicine (CAM) supplement, questionnaires used by prior studies, and chronic pain management guidelines. Definitions of complementary practices were based on descriptions provided in the National Health Interview Survey [13] and the National Center for Complementary and Integrative Health website [14]. Key informant interviews with 4 expert clinicians were used to refine health practice definitions.

One author (MAP) conducted cognitive interviews using a preliminary version of the HPI with 5 participants. Participants were mailed a questionnaire and instructed to complete it in one sitting, marking any items that were confusing or raised questions. Subsequently, semi-structured cognitive interviews were conducted to gather in-depth information about participants’ responses, following published recommendations [15, 16]. To assess whether they answered questions as intended by the developers, participants were instructed to “think aloud” as they completed the inventory. The cognitive interviewing process largely confirmed comprehension and clarity of items. Only minor changes were made after the cognitive interviews.

The final HPI covers 19 distinct health practices, each accompanied by a brief description (Additional file 1: Figure S1, for full text of the HPI including descriptions of practices). Questions ask about use of each practice in the past year. For each endorsed practice, follow-up questions ask about reasons for use (improve well-being/general health, manage pain, or manage a condition other than pain) and frequency of use in the past month (not at all, several days, more than half the days, nearly every day).

### Procedures and participants

Study participants were members of the Readiness and Resilience in National Guard Soldiers (RINGS) [17] longitudinal cohort study, which was originally designed to identify predictors of post-deployment health outcomes. Eligible cohort participants included 3890 Army National Guard Soldiers who were deployed to Iraq, Afghanistan, or Kuwait between 2006 and 2011, completed a baseline assessment before or during deployment, and completed at least 1 follow-up assessment post-deployment.

Data were collected from October 2015 to September 2016 using standard multiple-contact mailed survey methodology [18]. A questionnaire, cover letter, and $20 incentive were mailed to 3833 participants (52 others had untrackable addresses, 1 was incarcerated, and 4 were deceased). A postcard reminder and 2 additional survey mailings were sent to non-responders at 2-week intervals, with the final mailing delivered by priority mail. The overall response rate was 48.3% (*n* = 1850). The non-responders were slightly younger than responders and less likely to be female, but otherwise similar (see Additional file 2: Table S1, for characteristics of responders and non-responders). All study procedures were approved by the institutional review boards of the Minneapolis VA Health Care System and University of Minnesota. A waiver of documentation of informed consent was approved by both IRBs.

### Measures

Data for this study are cross-sectional and obtained from the 2015–2016 RINGS cohort follow-up survey described above. The mailed questionnaire included the HPI described above and measures assessing deployment experiences, pain, quality of life, mental health, substance use, and personality characteristics. For this analysis, variables were selected based on previously-demonstrated or hypothesized associations with non-pharmacological or complementary health practice use.

#### Demographics

Age at time of survey mailing was recorded from administrative records. Participants self-reported gender, race, ethnicity, educational attainment, employment, and length of military service.

#### Pain

Pain is a common reason for use of both complementary and conventional non-pharmacological therapies. The National Pain Strategy population health [19] pain persistence item (5 response version) was used to define chronic pain as the presence of pain on at least half the days in the previous 6 months. Pain severity was measured with the 3-item Pain, Enjoyment of life, General Activity (PEG) scale [20]. The 3 items ask participants to rate on a 0 to 10 scale over the past week their average pain severity, pain interference with enjoyment of life, and pain interference with general activity. The PEG has good responsiveness [21] and concurrent validity [20].

#### Military deployment experiences

To assess past combat exposure, participants were asked, “During any deployment, were you ever a participant or observer in direct combat operations?” They could respond, “Yes, participated in direct combat operation(s)”; “yes, observed or witnessed combat operation(s) but not participated”; “no.” To assess deployment injuries, participants were asked, “Were you wounded or injured during any deployment?” (response options, yes or no).

#### Self-reported health

Overall health was self-reported using the single-item global heath and 1-year retrospective global health questions from the Veterans RAND 12 Item Health Survey (VR-12) [22, 23]. Participants were asked, “In general would you say your health is: excellent, very good, good, fair or poor?” Overall health was dichotomized as excellent/very good versus good/fair/poor. For the retrospective questions, participants were asked, “Compared to one year ago, how would you rate your… Physical health in general now?” and “Emotional problems (such as feeling anxious, depressed or irritable) now?” Participants could respond, “much worse”, “slightly worse”, “about the same”, “slightly better”, or “much better.” These items were dichotomized as slightly worse/much worse versus much better/slightly better/about the same.

#### Mental health

Anxiety, depression, and poor self-rated health may be more prevalent in people who use complementary approaches than those who do not. [10, 24] Anxiety symptoms were measured using the Patient Reported Outcomes Measurement Information System (PROMIS) short form 8a anxiety scale [25]. The scale was dichotomized at 22 (corresponding to a T-score > 60) [26], consistent with moderate or severe anxiety. Depressive symptoms were measured with the 8-item Patient Health Questionnaire depression scale (PHQ-8) [27]. The PHQ-8 was dichotomized at 10 [27], consistent with moderate or severe depression. Posttraumatic stress symptoms were measured with the PTSD Checklist-5 (PCL-5) [28]. The PCL-5 was dichotomized at 33, consistent with probable PTSD [28].

#### Substance use

Alcohol use was measured with the Alcohol Use Disorders Identification Test (AUDIT) [29]. The AUDIT score was dichotomized above 7, consistent with problem alcohol use [29]. Illicit drug use was measured with the Drug Abuse Screening Test (DAST) [30]. A score above 0 represents any illicit drug use in the previous year.

#### Personality

Absorption (the tendency to be open to experiences and mindful states), is 1 of 11 primary traits measured by the Multidimensional Personality Questionnaire [31, 32]. Absorption has been shown to be positively associated with use of complementary non-pharmacological therapies [33]. Absorption was measured using the 12-item absorption subscale from the Multidimensional Personality Questionnaire-Brief Form [31, 32].

### Statistical analyses

We used latent class analysis (LCA) to identify distinct subgroups of users of health practices. LCA is an exploratory data reduction technique that categorizes participants into multiple discrete, non-overlapping classes based on similar patterns of observed data. Because the classes are latent, they cannot be directly observed and can only be estimated using observed response patterns. The purpose of the LCA in this study was to combine the HPI responses to the 19 practices into a small number of substantively meaningful classes about which inferences could be made. For an LCA with binary data, as in this study, the model estimates the probability that a member of each class endorses each item (i.e. each health practice). Estimates from the latent class model were used to calculate the probability an individual was in a class as a function of their actual response pattern.

Frequency of health practice use was dichotomized as any use versus no use in the previous 12 months. Fewer than 2% of participants reported using biofeedback, Tai Chi/Qi Gong, Healing Touch/Reiki, homeopathy, and hypnotherapy; including these rare approaches led to estimability problems, so they were excluded from the LCA. The latent class model used only self-report of individual health practices to predict class membership. Separate models were fit with 1 to 11 latent classes. The fit of these models was compared using the Bayesian Information Criterion and Akaike’s Information Criterion to determine the best fitting number of classes. Participants were assigned to classes based on maximum posterior probability.

To examine associations of participant characteristics with latent class membership, selected variables (described above) were used as predictors in a multinomial regression model with the latent classes as outcomes. Marginal effects (i.e. difference in class membership probabilities) were calculated from the regression results by standardizing to the distribution of covariates in the total sample and calculating the difference in probability of class membership between levels of the covariate [34]. All analyses were performed in Stata 15 [35].

### Missing data

Approximately 14% of participants had missing data for at least 1 predictor variable in the multinomial regression model. To address the concern that this missingness could bias results, we imputed 20 datasets by chained multiple imputation to allow all participants to be included [36–38]. Continuous measures and ordered scales were imputed by predictive mean matching with 5 nearest neighbors and imputed values were drawn from 20 independent bootstrap samples [39, 40]. Continuous measures were dichotomized after imputation. Binary and factor variables were imputed by logistic regression or multinomial logistic regression. All imputed variables were included in the chained equations and maximum probability latent class was included as a fixed (not imputed) variable. The scales that were not included in the multinomial logistic regression were still included in the chained imputation equations to improve performance of the imputation.

## Results

Table 1 presents demographic characteristics of participants and mean scores on self-report scales. Participants were mostly male and white, with a mean age of 39 years (SD = 9); 41% had chronic pain and over 20% screened positive for mental health problems (e.g., depression, anxiety, PTSD).

**Table 1:** Characteristics of Minnesota National Guard Veterans who responded to the mailed survey conducted from October 2015 to September 2016 (*N* = 1850)

Characteristic	Mean (SD) or % (N)
Male, % (N)	90.3% (1668)
Age, Mean (SD)	38.7 (9.2)
White, % (N)	90.1% (1656)
Obtained 4-year degree, % (N)	42.6% (772)
Injured on deployment, % (N)	27.0% (493)
Pain	
Chronic pain, % (N)	41.2% (749)
Intensity and interference (PEG), Mean (SD)	2.4 (2.4)
Self-rated health	
Current health excellent/very good^a^, % (N)	43.1% (796)
Mental health	
Anxiety at least moderate (PROMIS-Anxiety 8a ≥ 22), % (N)	21.8% (400)
Depression (PHQ-8 ≥ 10), % (N)	21.7% (392)
Probable PTSD (PCL-5 ≥ 33), % (N)	19.5% (339)
Problem alcohol use (AUDIT ≥8), % (N)	21.6% (394)
Past year illicit drug use (DAST > 0), % (N)	10.3% (184)
Absorption (MPQ-BF Absorption, T-scores), Mean (SD)	47.6 (10.8)

*Abbreviations: PEG* 3-item PEG scale, *VR-12* Veterans RAND 12-Item Health Survey

^a^Current health excellent/very good vs good/fair/poor; VR-12 overall health item

Table 2 summarizes HPI responses of all participants. Complete data about past-year use of all 19 health practices were available for 1817 participants (98%). Twenty-five participants (1%) completely skipped HPI past-year use items; 6 (< 1%) skipped past-year use for 1 of the modalities; and 2 (< 1%) skipped several of the past-year items. Practices commonly used for pain tended to be practitioner-delivered, including acupuncture, chiropractic, massage and manual physical therapy. Practices used for well-being or general health tended to be active, self-directed practices, including yoga, meditation/mindfulness, aerobic exercise and strengthening/stretching exercise.

**Table 2:** Self-reported past-year use of Health Practices Inventory approaches by Minnesota National Guard Veterans (*N* = 1825), who participated in the mailed survey conducted from October 2015 to September 2016

Health practice^a^	%	*n*	Reason for use^b^						Past month frequency of use^c^					
			Well-being/ general health		Treat pain		Treat another condition		None		Several days		More	
			%	*n*	%	*n*	%	*n*	%	*n*	%	*n*	%	*n*
Acupuncture	5%	86	37%	32	72%	62	22%	19	69%	59	28%	24	0%	0
Biofeedback	2%	30	70%	21	13%	4	17%	5	47%	14	33%	10	13%	4
Chiropractic	32%	578	31%	179	83%	477	8%	47	46%	268	46%	266	2%	14
Massage	24%	431	52%	226	63%	272	8%	35	56%	242	33%	144	2%	9
Manual physical therapy	13%	242	15%	37	80%	193	12%	30	41%	100	43%	105	8%	20
Healing Touch/Reiki	1%	21	52%	11	52%	11	38%	8	48%	10	43%	9	5%	1
Hypnotherapy	1%	9	33%	3	22%	2	44%	4	67%	6	0%	0	22%	2
Psychotherapy	13%	233	54%	125	9%	22	50%	116	39%	91	46%	107	5%	12
Support groups	4%	79	62%	49	8%	6	39%	31	30%	24	46%	36	13%	10
Spiritual/traditional healing system	6%	115	78%	90	14%	16	29%	33	10%	11	39%	45	44%	51
Relaxation	18%	335	63%	212	19%	65	37%	125	13%	42	54%	182	25%	84
Meditation/Mindfulness	10%	190	74%	140	12%	22	32%	60	10%	19	52%	99	25%	47
Yoga	10%	174	85%	148	33%	58	14%	24	31%	54	50%	87	8%	14
Tai Chi/Qi Gong	1%	15	87%	13	33%	5	7%	1	27%	4	33%	5	13%	2
Strength/stretch exercise	44%	799	76%	604	34%	269	10%	78	9%	69	37%	296	45%	360
Aerobic exercise	36%	661	88%	580	12%	80	9%	56	4%	28	42%	278	45%	300
Diet	7%	120	77%	92	21%	25	19%	23	9%	11	18%	21	63%	76
Herbal supplement	10%	175	80%	140	15%	27	19%	34	5%	8	26%	46	60%	105
Homeopathy	1%	25	60%	15	44%	11	40%	10	36%	9	32%	8	28%	7

The practices are listed in the order they appear in the survey (Additional file 1: Figure S1, for full text of the Health Practices Inventory)

^a^The Health Practices Inventory approaches are grouped based on the a priori categorization described in the text; a summary variable was created for each of the groupings indicating use of any of the modalities in that group

^b^Participants who reported using a Health Practices Inventory modality were asked to identify their reason(s) for using it; the reasons are not mutually exclusive and they could pick any combination of the 3 reasons listed or none at all

^c^Participants who reported using a Health Practices Inventory modality were asked to identify the frequency they used it in the past month; these frequencies are mutually exclusive but do not always add up to 100% because of missingness

### Classes of health practice users

The best-fitting latent class model had 6 distinct and substantively meaningful classes (see Additional file 3: Table S2, for model fit statistics; see Additional file 4: Table S3, for characteristics of class members). Table 3 presents the prevalence of use of the HPI modalities within the 6 latent classes, which were labeled to reflect the distinguishing prevalence of modalities between classes. One “low use” class represented very low rates of health practice use (50% of participants, *n* = 923). Five classes represented greater use of health practices compared to the low use class. The “exercise” class (23%, *n* = 426) had high rates of aerobic exercise (used by 86% of class members) and strength/stretching exercise (86%) and lower use of other health practices compared to the total sample. The “psychotherapy” class (6%, *n* = 112) was the only class with high use of psychotherapy and of support groups, and also had high use of mindfulness and relaxation. The “manual therapies” class (12%, *n* = 213) had high use of practitioner-delivered manual therapies, including chiropractic, massage, and acupuncture, and moderate use of exercise practices. The “mindfulness” class (5%, *n* = 101) had high use of relaxation practices, mindfulness and yoga, and moderate use of exercise. The “multimodal” class (4%, *n* = 75) was the smallest class and had high use of every modality except psychotherapy and support groups. The 6 classes identified in the best-fit latent class model were robust across other latent class model solutions with different numbers of classes. Notably, the low use, exercise and psychotherapy classes were easy to identify in the next best-fit models (5 and 7 classes). Major distinctions between latent class models related to differential use of specific complementary health practices between classes. With 7 classes, the manual therapies class was split into 2 classes, a class with high rates of strengthening/stretching and aerobic exercise and a class with low rates of exercise. With 5 classes, the mindfulness and multimodal classes merged into one class.

**Table 3:**
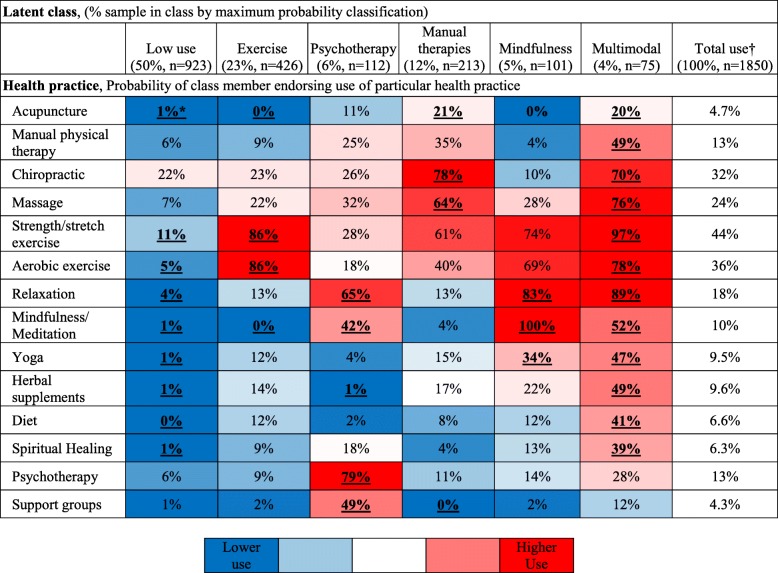
Probability of use of Health Practices Inventory approaches within latent classes of 1850 Minnesota National Guard Veterans who participated in a mailed survey conducted from October 2015 to September 2016.

Health practices were listed in an order that maximized the visual clustering of modalities with similar use within classes

*Bolding added to highlight proportions that distinguish classes; all proportions that represent an odds ratio greater than 5 compared to use among all responders were considered much higher use than in the total sample and were **bolded**; all proportions that represent an odd ratio less than 0.2 compared to use among all responders were considered much lower use than in the total sample and were ***bolded and italicized***

†Total use represents the proportion of use of modalities in the total sample. This is not a latent class

Use of complementary approaches varied among the classes in the final model, as measured by the number of different complementary practices endorsed (range: 0 to 12). Compared with 31% of members of the low use class, 100% of members of the manual therapies, mindfulness, and multimodal classes reported use of at least 1 complementary health practice. The distributions were highly skewed. The multimodal class had the highest overall use of complementary approaches (median = 5; SE: 0.1). The manual therapies class (median = 2; SE: 0.06), mindfulness (median = 2; SE: 0.1) and psychotherapy (median = 2; SE: 0.1) classes had similar moderate use of complementary approaches. The exercise (median = 1; SE: 0.04) and low use (median = 0; SE: 0.02) classes had the least.

### Associations of participant characteristics with health practice class

**Fig. 1:**
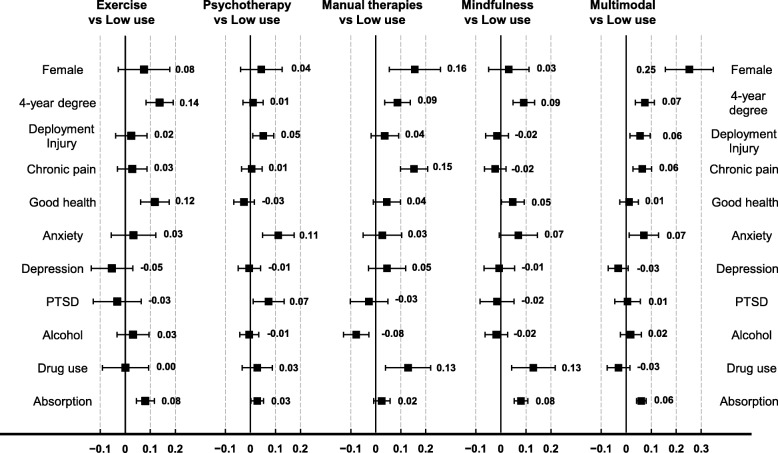
Prevalence differences of class membership due to covariates; results of multinomial logistic regression from Minnesota National Guard Veterans who participated in the mailed survey conducted from October 2015 to September 2016. Interpreted as the difference in risk (probability) of membership in this latent class between levels of the covariate where a negative value means lower probability of membership in this class compared to probability at the reference level of the covariate; other covariates are standardized to their distribution among all responder. All covariates are dichotomous except for absorption, which is continuous. For absorption, a one-unit change represents a 2-point change on the absorption scale, equivalently a 7-point change on the T-score

Figure 1 presents estimates of the effect of each covariate on probability of membership in each of the latent classes, compared with low use. A positive value means that the covariate increases the probability of membership in that latent class compared to the low use class. For example, being female was associated with a 0.25 greater prevalence of the multimodal class relative to the low use class. Also, a positive screen for problem alcohol use instead of a negative screen was associated with a 0.08 decrease in the prevalence of the manual therapies class compared to the low use class. Female sex was positively associated with membership in the multimodal class and problem alcohol use was negatively associated with membership in the manual therapies class, compared to the low use class. (see Additional file 5: Table S4, for effects with confidence intervals in tabular form.)

Some demographic characteristics predicted higher rates of health practice use in general, while others distinguished between specific classes. Higher absorption and higher education were associated with higher prevalence of the 5 health practice use classes (compared to the low use class). Depression was not associated with any of the HPI-use classes. PTSD was only associated with the psychotherapy class. Problem alcohol use was only (negatively) associated with the manual therapies users class.

Each class had a unique set of covariates that were associated with a difference in prevalence compared to the low use class. For example, higher anxiety was associated with the psychotherapy class and the multimodal class, but higher anxiety and higher PTSD distinguished the psychotherapy class from the multimodal class. Chronic pain was associated with the manual therapies class and the multimodal class; however, chronic pain with higher absorption distinguished the multimodal class from the manual therapies class. Both the manual therapies and the mindfulness classes were associated with higher education and higher illicit drug use; however, the manual therapies class was distinguished by being female and having chronic pain, whereas the mindfulness users class was distinguished by higher absorption and better self-rated health.

## Discussion

This study found that integrated patterns of complementary and conventional approaches identify unique classes of health practice users. These classes had unique sets of predictors. For example, the exercise class was characterized by better self-rated health, higher educational attainment, and more open and mindful personality (absorption), while the psychotherapy class was characterized by higher psychological distress compared to the low use class. These data were collected using a novel instrument, the HPI. The HPI is an efficient way to collect self-report data on individuals’ use of multiple complementary and conventional non-pharmacological therapies with minimal missing data.

Results from this study, which used LCA to explore data-driven patterns of use of health approaches, are broadly consistent with prior classifications based on qualitative research and expert opinion. In this study, active approaches, such as exercise, yoga, and mindfulness, tended to cluster together (Table 3). Similarly, practitioner-delivered approaches, such as chiropractic, massage, or manual physical therapy, tended to cluster together. Authors of the National Health Interview Survey CAM supplement previously noticed a distinction between active and practitioner-delivered practices [41]. Through qualitative interviews, they found participants experienced active practices differently than practitioner-delivered practices [41]. This distinction has persisted in the CAM supplement questions. In other instances, practices are classified based on expert opinion. Categorizations differ between experts. The National Center for Complementary and Integrative Health (NCCIH) has broadly classified practices as Mind and Body, Natural Products, and Others [42]. On the other hand, authors from the National Center for Health Statistics (NCHS) have classified approaches as Natural Products, Practitioner-based, Mind and Body, or Whole Medical Systems [43]. Even the categories that share the same name do not contain the same list of practices (e.g. chiropractic and acupuncture are “Mind and Body” in the NCCIH taxonomy but chiropractic is “Practitioner-based” and acupuncture is “Whole Medical Systems” in the NCHS taxonomy).

Classifying practices based on practitioner-delivered versus active/self-directed emerged in the patterns we observed in the present study. Our categorization were based on real-world patterns of use, instead of expert opinion or practitioner experience. These 3 different approaches to classifying nonpharmacological health practices (i.e. categorization by expert opinion, categorization based on experiences of health practice users informed by qualitative interview, and latent class statistical categorization based on observed patterns of use) all triangulate towards similar, meaningful categories of health practices. Convergence of findings using several different approaches adds external validity to the emergent classes of the present analysis.

The distinction between active and practitioner-delivered practices appears to be functionally important. This study found differences in the reported reasons for using practices from these 2 categories. Participants were more likely to report using practitioner-delivered approaches for pain rather than well-being, and far more likely to report using active approaches for well-being rather than pain. In fact, the practices most-often used for wellness were aerobic exercise, yoga, and tai chi/qi gong, and the practices most-often used for pain were manual physical therapy, chiropractic, and acupuncture. Despite this difference in reason for use, practitioner-delivered practices have not been shown to be superior to active practices for pain [44–46].

Prior studies have found that use of complementary health approaches is greater among women, middle age groups, people with more education and higher income, and people reporting a musculoskeletal pain disorder [2, 43]. Our results are largely consistent with prior studies; higher use of non-pharmacological practices generally was predicted by female sex and higher educational attainment. Higher absorption has been shown to be associated with higher use of complementary approaches [33], although our results suggest this may apply more to active than practitioner-delivered practices. We observed that the predictors of use are quite variable within classes.

This study has several limitations. First, although the HPI allows collection of detailed data about how often and why participants use non-pharmacological therapies, we dichotomized health practice use as any versus no use in the past year, which statistically equates daily use and one-time use. We did this because when we included frequency in our LCA the groups seemed to be defined by frequency of use of the exercise practices, which did not suit the purposes of this study. Frequency of exercise practices may have dominated because they were the most common practices and variability in exercise frequency was large. A second limitation is the small prevalence of some classes (e.g., multimodal). Although there is no consensus on adequate class size, estimability problems due to small class prevalence are diminished in large total sample sizes (500 or 1000) [47]. Furthermore, model fit statistics supported the model we chose as best fit for these data. Third, the overall response rate was 48.3% for the follow-up survey; however, non-responders were not substantially different from responders, and the response rate is reasonable considering some cohort members have been followed for 10 years already. Fourth, there was a small amount of item missingness. We used multiple imputation to address this and results were consistent with complete case analysis. Fifth, participants were National Guard veterans, whose characteristics and health practice behaviors likely differ from the general population; therefore, it is important these findings be replicated in other samples.

## Conclusions

The practical categorization of health practices as active or practitioner-delivered emerged in the distinct patterns of use identified in this sample of recently deployed veterans. Our analyses show the value of considering integrated use of health practices. There are important similarities among individuals within latent classes of use that would be obscured by collapsing all users of individual practices or collapsing all users of complementary practices. Because individuals use multiple health practices that may have overlapping effects, it may be important to consider overlapping effects in studies of individual health practices. Our findings should be investigated in other contexts and with other samples. In particular, latent class analyses with new samples could provide evidence for or against these 6 classes.

## Additional files

Additional file 1: Supplemental Digital Content 1.pdf. (PDF 247 kb)

Additional file 2: Supplemental Digital Content 2.pdf. (PDF 35 kb)

Additional file 3: Supplemental Digital Content 3.pdf. (PDF 39 kb)

Additional file 4: Supplemental Digital Content 4.pdf. (PDF 15 kb)

Additional file 5: Supplemental Digital Content 5.pdf. (PDF 60 kb)

## Abbreviations

AUDIT: Alcohol Use Disorders Identification Test
CAM: Complementary and Alternative Medicine
DAST: Drug Abuse Screening Test
HPI: Health Practices Inventory
LCA: Latent class analysis
NCCIH: National Center for Complementary and Integrative Health
NCHS: National Center for Health Statistics
PCL-5: PTSD Checklist, DSM-V criteria
PEG: Pain, Enjoyment of life, General Activity [pain scale]
PHQ-8: 8-item Patient Health Questionnaire [depression scale]
PROMIS: Patient Reported Outcomes Measurement Information System
RINGS: Readiness and Resilience in National Guard Soldiers [study]
VA: [Department of] Veterans Affairs
VR-12: Veterans RAND 12-item Health Survey
