# Supplementary material for: Patterns of conventional and complementary non-pharmacological health practice use by US military veterans: a cross-sectional latent class analysis
Source: BMC Complement Altern Med. 2018 Sep 5;18:246. doi: 10.1186/s12906-018-2313-7 (PMC6125945; doi:10.1186/s12906-018-2313-7)
Supplement: Supplementary file 1 — Supplemental Digital Content 1.pdf. (PDF 247 kb) [file 12906_2018_2313_MOESM1_ESM.pdf]

Below is a list of treatments, practices, and services that people use for health reasons. Please tell us if you have used each one in the past year by marking "Yes" or "No." If you mark "Yes," please tell us why and how often you have used the treatment.

In the past year, I have used...		**If yes, why?** Improve well-being/ general health	Manage pain	Manage a condition other than pain	**How often in the past month?** Not at all	Several days	More than half the days	Nearly every day
		Choose all that apply.						
**Acupuncture:** Stimulation of specific points of the body, with thin needles.	○ Yes → ○ No	○	○	○	○	○	○	○
**Biofeedback:** Use of sensors to teach control over body functions such as breathing, heart rate and muscle tone.	○ Yes → ○ No	○	○	○	○	○	○	○
**Chiropractic:** Hands on adjustment of spine and joints to improve alignment, function, or pain.	○ Yes → ○ No	○	○	○	○	○	○	○
**Massage Therapy:** Hands on pressure, rubbing, or manipulation of muscles and soft tissues.	○ Yes → ○ No	○	○	○	○	○	○	○
**Rehabilitation Therapies:** Hands on treatment such as traction, TENS, ultrasound, or mobilization by a PT or OT to treat pain or injury.	○ Yes → ○ No	○	○	○	○	○	○	○
**Healing/Therapeutic Touch or Reiki:** Gentle techniques to alter energy fields and restore balance to allow healing.	○ Yes → ○ No	○	○	○	○	○	○	○
**Hypnosis/Hypnotherapy:** Use of deep relaxation and power of suggestion to change responses and behaviors.	○ Yes → ○ No	○	○	○	○	○	○	○
**Psychotherapy:** One-on-one or group talk therapy, such as cognitive behavioral therapy (CBT), by a psychologist or other mental health provider.	○ Yes → ○ No	○	○	○	○	○	○	○
**Support Group:** A therapeutic group formed around a shared problem or experience.	○ Yes → ○ No	○	○	○	○	○	○	○
**Spiritual or Traditional Healing:** Use of faith-based, religious, or spiritual practice to promote health or healing.	○ Yes → ○ No	○	○	○	○	○	○	○

Below is a list of treatments, practices, and services that people use for health reasons. Please tell us if you have used each one in the past year by marking "Yes" or "No." If you mark "Yes," please tell us why and how often you have used the treatment.

In the past year, I have used...		If yes, why? Improve well-being/ general health	Manage pain	Manage a condition other than pain	How often in the past month? Not at all	Several days	More than half the days	Nearly every day
		Choose all that apply.						
**Relaxation Techniques:** Use of breathing, guided imagery, or progressive muscle relaxation to cause a relaxation response.	○ Yes → ○ No	○	○	○	○	○	○	○
**Meditation/Mindfulness Practice:** Use of focused attention and non-judgmental awareness, including transcendental, mindfulness meditation, Mindfulness Based Stress Reduction.	○ Yes → ○ No	○	○	○	○	○	○	○
**Yoga:** Practices that combine physical postures, breathing techniques, and meditation or relaxation.	○ Yes → ○ No	○	○	○	○	○	○	○
**Tai Chi/Qi Gong:** Combined practice of slow movements, coordinated-breathing, and mental focus.	○ Yes → ○ No	○	○	○	○	○	○	○
**Stretching/Strengthening Exercise Therapy:** Training or home exercise program using stretches or weights to improve flexibility, posture, or strength.	○ Yes → ○ No	○	○	○	○	○	○	○
**Aerobic Exercise Therapy:** Training or home exercise program using activities such as walking, swimming or aerobics to increase fitness or edurance.	○ Yes → ○ No	○	○	○	○	○	○	○
**Diet-based Therapies:** Use of specific diet for health purposes, such as anti-inflammatory.	○ Yes → ○ No	○	○	○	○	○	○	○
**Herbal Supplements:** Use of plant-based, herb, or botanical product such as echinacea.	○ Yes → ○ No	○	○	○	○	○	○	○
**Homeopathy:** Individualized treatment by a homeopathic practioner or use of homeopathic remedies.	○ Yes → ○ No	○	○	○	○	○	○	○
