# Supplementary material for: Patterns of conventional and complementary non-pharmacological health practice use by US military veterans: a cross-sectional latent class analysis
Source: BMC Complement Altern Med. 2018 Sep 5;18:246. doi: 10.1186/s12906-018-2313-7 (PMC6125945; doi:10.1186/s12906-018-2313-7)
Supplement: Supplementary file 2 — Supplemental Digital Content 2.pdf. (PDF 35 kb) [file 12906_2018_2313_MOESM2_ESM.pdf]

**Characteristic**	**Responders**	**Non-responders**
Male, % (N)	90.5 (1638)	92.1 (1744)
Age, Mean (SE)	38.7 (9.2)	33.5 (7.9)
White, % (N)	93.8 (1592)	92.9 (1658)
Lives in urban area, % (N)	51.6 (933)	52.0 (985)
Eligible for VA healthcare, % (N)	59.2 (1071)	51.9 (982)
Anxiety-related diagnosis from VA within past year, % (N)	9.4 (174)	11.4 (225)
Depression-related diagnosis from VA within past year, % (N)	11.2 (208)	11.9 (235)
PTSD diagnosis from VA within past year, % (N)	13.0 (242)	11.7 (231)
