# Supplementary material for: Patterns of conventional and complementary non-pharmacological health practice use by US military veterans: a cross-sectional latent class analysis
Source: BMC Complement Altern Med. 2018 Sep 5;18:246. doi: 10.1186/s12906-018-2313-7 (PMC6125945; doi:10.1186/s12906-018-2313-7)
Supplement: Supplementary file 3 — Supplemental Digital Content 3.pdf. (PDF 39 kb) [file 12906_2018_2313_MOESM3_ESM.pdf]

No. classes	LL	AIC	aBIC	BIC	CAIC	S	% success
1	-10165	20357	20390	20435	20449	1.000	100%
2	-9416	18890	18957	19049	19078	0.707	100%
3	-9206	18498	18598	18735	18778	0.597	98%
4	-9111	18337	18472	18657	18715	0.517	39%
5	-9020	18186	18356	18588	18661	0.429	91%
6	-8971	18110	18306	18573	18657	0.455	42%
7	-8943	18071	18288	18583	18676	0.387	31%
8	-8918	18039	18277	18601	18703	0.372	1%
9	-8893	18020	18293	18665	18782	0.313	1%
10	-8873	18002	18300	18707	18835	0.222	2%
11	-8851	17971	18283	18709	18843	0.164	1%

Abbreviations: LL: Log-likelihood; AIC: Akaike's Information Criterion; aBIC: Adjusted Bayesian Information Criterion; BIC: Bayesian Information Criterion; CAIC: Bozdogan's consistent AIC; S: Entropy; % success: proportion of random seed values that converged to the global minimum log-likelihood for that number of classes.
