# Supplementary material for: Patterns of conventional and complementary non-pharmacological health practice use by US military veterans: a cross-sectional latent class analysis
Source: BMC Complement Altern Med. 2018 Sep 5;18:246. doi: 10.1186/s12906-018-2313-7 (PMC6125945; doi:10.1186/s12906-018-2313-7)
Supplement: Supplementary file 4 — Supplemental Digital Content 4.pdf. (PDF 15 kb) [file 12906_2018_2313_MOESM4_ESM.pdf]

**Latent class**, (% sample in class by maximum probability classification)						
	Low use (50%, n=923)	Exercise (23%, n=426)	Psychotherapy (6%, n=112)	Manual therapies (12%, n=213)	Mindfulness (5%, n=101)	Multimodal (4%, n=75)
**Health practice**, Probability of class member endorsing use of particular health practice, % or Mean						
Overall health fair/poor	13.8%	11.0%	21.0%	12.4%	12.9%	10.5%
Physical health worse	28.1%	27.6%	34.5%	25.2%	23.5%	33.8%
Emotional problems worse	24.7%	21.4%	49.2%	23.1%	29.9%	30.4%
PROMIS-Anxiety	14.8	15.2	21.5	14.8	17.4	16.9
PHQ-8	5.4	5.3	9.0	5.9	6.1	5.8
PCL-5	15.8	15.8	30.7	16.4	18.8	18.6
DAST > 0	8.6%	9.4%	12.6%	14.6%	18.6%	6.7%
AUDIT	5.1	5.5	6.7	4.6	5.1	6.1
PAM	38.9	40.2	38.8	40.4	40.0	42.4
MMPI - Absorption	4.4	5.1	5.3	4.9	6.6	6.7
