# Supplementary material for: Patterns of conventional and complementary non-pharmacological health practice use by US military veterans: a cross-sectional latent class analysis
Source: BMC Complement Altern Med. 2018 Sep 5;18:246. doi: 10.1186/s12906-018-2313-7 (PMC6125945; doi:10.1186/s12906-018-2313-7)
Supplement: Supplementary file 5 — Supplemental Digital Content 5.pdf. (PDF 60 kb) [file 12906_2018_2313_MOESM5_ESM.pdf]

Exercise vs. Low use	Psychotherapy vs. Low use	Manual therapies vs. Low use	Mindfulness vs. Low use	Multimodal vs. Low use
**Female,** RD (95% CI)*				
0.08 (−0.03 to 0.18)	0.04 (−0.04 to 0.13)	0.16 (0.05 to 0.26)	0.03 (−0.05 to 0.11)	0.25 (0.16 to 0.35)
**4-year degree,** RD (95% CI)				
0.14 (0.08 to 0.19)	0.01 (−0.03 to 0.05)	0.09 (0.04 to 0.14)	0.09 (0.05 to 0.13)	0.07 (0.04 to 0.11)
**Injured during deployment,** RD (95% CI)				
0.02 (−0.04 to 0.09)	0.05 (0.01 to 0.09)	0.04 (−0.02 to 0.09)	−0.02 (−0.06 to 0.03)	0.06 (0.02 to 0.10)
**Chronic Pain,** RD (95% CI)				
0.03 (−0.03 to 0.09)	0.01 (−0.04 to 0.05)	0.15 (0.10 to 0.21)	−0.02 (−0.07 to 0.02)	0.06 (0.03 to 0.10)
**Self-rated health Excellent/Very Good,** RD (95% CI)				
0.12 (0.06 to 0.18)	−0.03 (−0.07 to 0.02)	0.04 (−0.01 to 0.10)	0.05 (0.00 to 0.09)	0.01 (−0.03 to 0.05)
**Mild or greater anxiety,** RD (95% CI)				
0.03 (−0.06 to 0.12)	0.11 (0.05 to 0.17)	0.03 (−0.05 to 0.10)	0.07 (−0.01 to 0.15)	0.07 (0.01 to 0.13)
**Depression,** RD (95% CI)				
−0.05 (−0.14 to 0.03)	−0.01 (−0.05 to 0.04)	0.05 (−0.03 to 0.12)	−0.01 (−0.07 to 0.05)	−0.03 (−0.07 to 0.01)
**PTSD,** RD (95% CI)				
−0.03 (−0.13 to 0.06)	0.07 (0.01 to 0.13)	−0.03 (−0.10 to 0.05)	−0.02 (−0.08 to 0.05)	0.01 (−0.05 to 0.06)
**Problem alcohol use,** RD (95% CI)				
0.03 (−0.03 to 0.10)	−0.01 (−0.04 to 0.03)	−0.08 (−0.13 to −0.03)	−0.02 (−0.07 to 0.03)	0.02 (−0.02 to 0.06)
**Any illicit drug use,** RD (95% CI)				
0.00 (−0.09 to 0.09)	0.03 (−0.03 to 0.09)	0.13 (0.04 to 0.22)	0.13 (0.04 to 0.22)	−0.03 (−0.08 to 0.01)
**Absorption,** † RD (95% CI)				
0.08 (0.04 to 0.12)	0.03 (0.00 to 0.05)	0.02 (−0.01 to 0.06)	0.08 (0.05 to 0.11)	0.06 (0.04 to 0.08)

* Interpreted as the difference in risk (probability) of membership in this latent class between levels of the covariate where a negative value means lower probability of membership in this class compared to probability at the reference level of the covariate; other covariates are standardized to their distribution among all responder

† Continuous, a one-unit change represents a 2-point change on the absorption scale, equivalently a 7-point change on the T-score scale
